# N-Methyl-d-aspartate (NMDA) receptor antibody in relation to autism spectrum disorder (ASD): presence and association with symptom profile

**DOI:** 10.1186/s43045-021-00141-5

**Published:** 2021-11-01

**Authors:** Heba Hamed Elshahawi, Ghada Refaat Amin Taha, Hanan Mohamed Ezzeldin Azzam, Reem H. El Ghamry, Ahmed Adel Mohammad Abdelgawad, Mohammad Abdullah Ahmad Abdullah Elshiekh

**Affiliations:** Ain Shams University, 38 Abbasia square, Cairo, 1181 Egypt

**Keywords:** Autism, Autoimmune, Autoantibodies, NMDA receptor

## Abstract

**Background:**

Several studies pointed to immune dysregulation abnormalities linked to autism spectrum disorders (ASD). Of those, several autoantibodies had been identified. Recent findings of N-methyl d-aspartate (NMDA) antibodies in autoimmune encephalitis suggested that it caused symptoms like autistic regression. Thus, the purpose of the study was to test for the presence of anti-NMDAR antibodies in the ASD disorder population and to correlate this with the clinical findings.

**Results:**

Eighty-seven autistic children, 4–12 years old, were enrolled in the study and were matched with sixty typically developing children used as controls. The diagnosis of cases was confirmed by ADOS-2 and clinical evaluation. None of the control children had positive anti-NMDAR antibodies, while 26.4% (23 children) of the patients’ group were positive for serum anti-NMDA receptor antibodies (> 200 pg/ml, *p* = 0.0157). The positive anti-NMDAR antibody was statistically correlated with better speech stage (*p* = 0.017), more severe stereotyped behavior (*p* ≤ 0.001), and abnormal EEG findings (*p* = 0.025).

**Conclusions:**

There is a possibility of the presence of anti-NMDAR antibodies in the autism spectrum disorder population with certain characteristics, especially the severity of the stereotyped behaviors.

## Background

Autism spectrum disorder (ASD) is a pervasive developmental disorder characterized by impairment in communication and reciprocal social interaction, as well as stereotypic/repetitive behaviors. The etiology of autism is still unclear, but a collection of genetic, developmental, and environmental factors is most plausible [6].

About 10–20% of autism cases can be attributed to evident genetic causes, yet incomplete concordance rates for monozygotic twins and phenotypical variabilities indicate the likely influence of environmental risk factors in the development of ASD [9].

ASD has been linked to immunological dysregulation on many levels. Allergies and autoimmunity diagnoses were significantly more common in children with ASD [34]. Several studies have found an increased incidence of immunological disorders in children with ASD [18].

Some studies pointed out to associations between immune system abnormalities or cytokine aberrations with ASD [21]. Activated microglia and increased microglial density have been found in the dorsolateral prefrontal cortex of the autistic brain [23]. Evidence also included upregulation of inflammation markers [27].

Several autoantibodies have been identified in ASD. These included anti-BDNF, anti-endothelial cells, anti-MBP (myelin basic protein), anti-mitochondrial DNA, anti-ganglioside, anti-neuronal, ANA, anti-DNA, and anti-nucleosome autoantibodies [18]. Some systemic autoantibodies were also detected in ASD patients [22].

One of the studied systems in autism is the glutamate neurotransmitter system, particularly the NMDA receptor. It has been suggested that abnormalities in NMDA function lead to autistic-like behaviors in mice, which if corrected early leads to the prevention of autistic-like behaviors [8]. Furthermore, Anti-NMDA encephalitis has been linked to a picture of autistic regression in a case report of a toddler [28].

We hypothesize that there is a possibility of the presence of anti-NMDAR antibodies in ASD and could be related to the pathogenesis of ASD. Therefore, the objective of the current study was to test for the presence of anti-NMDAR antibodies in the ASD population and to correlate this with the clinical findings.

## Methods

The aim of the study was to search for the possible presence of anti-NMDA receptor antibodies in the autism population and identify its clinical correlates. The study was conducted in Ain Shams University hospitals, Cairo, Egypt, on patients attending child psychiatry clinics in the Okasha Institute of Psychiatry, neuropsychiatry department*.*

### Study design

A case-control design testing the presence of anti-NMDAR antibodies in sixty typically developing children and eighty-seven children with autism. Then, an analytical study was carried on data from the eighty-seven children with autism.

### Patients

A sample size of 100 participants was calculated to achieve 80% power to detect an effect size of 0.3000 using a 2 degrees of freedom chi-square test with a significance level (alpha) of 0.050 (confidence level: 95%) but the study had to be terminated early due to COVID-19 lockdown. Only the first 87 (confidence level: 83%) children with autism were enrolled in the study.

Both genders 4–12 years old, physically healthy, diagnosed clinically according to DSM-5 criteria and Autism Diagnostic Observation Scale (ADOS) to have ASD with stable concomitant biomedical treatments for at least 2 weeks before enrollment were recruited. A reliable caregiver agreeing to give consent to participate in the study was essential for enrollment.

Patients with a comorbid clinical diagnosis of ADHD, tic disorder, and psychotic disorders were ineligible to participate in the study.

### Controls

Both genders, typically developing children 4–12 years old and physically healthy were enrolled from children or siblings of children seeking medical advice for non-major illnesses (e.g., gastroenteritis and middle ear infections) from the pediatric department. Inquiry about developmental milestones was made and only children with adequate development in motor, language, and social milestones were recruited as controls.

### Ethical considerations

Study aim, objectives, and design were explained to the children’s caregivers and written informed consent was signed prior to enrollment in the study. They were also informed that they can withdraw from the study at any time without any negative effect on their relationship with the health care providers. The consent was done in accordance with the ethical guidelines of the ethical committee of the faculty of medicine, Ain Shams University. The code for the ethical approval committee for the study is FMASU M D 366/2018.

### Tools and procedures

1. *Clinical data*: Patients and controls were described regarding sociodemographic data and history of regression after normal development or not.
2. *Autism diagnostic observation schedule (ADOS), 2nd edition* [20]: It was used to verify the diagnosis of autism. The ADOS contains 5 modules to be used to diagnose patients, they are divided according to the age of the patient and speech stage. Those used in this study were the preverbal/single words module for children 3 years and older and phrase speech for cases.
3. *Arabic version of Vineland adaptive behavior scale* [10, 32]: The scores obtained from the 1st 4 subscales (communication, skills of daily living, social skills, and motor skills) were used for cases to obtain the total calibrated score from the scale. Only the first 4 subscales of the scale were used without including the fifth, the non-adaptive behavior, subscale. Another method for data presentation, the age coefficient, was used by the researchers. It is obtained by dividing the equivalent age of the child’s adaptive level by the actual chronological age.
4. *Arabic version of Gilliam Autism Rating Scale (GARS)* [1, 14].: It is used to determine the probability of ASD diagnosis. Although a screening tool, it was useful for symptom domain analysis as it takes mainly stereotyped behaviors and social domain into consideration. It was also used for cases only.
5. *Stereotype behavior scale for autism spectrum disorders* [2]: This scale is an Egyptian scale designed, tested, and calibrated by 2 professors in the faculty of special education, Cairo University. This scale measures the severity and degree of repetition of different types of stereotyped behaviors. This scale divides the stereotyped behaviors into sensory, verbal, motoric, emotional, and routinistic. It was used for cases only and only the severity part of the scale was used.
6. *Qualitative digital Electro-encephalo gram (EEG*): A ()min qualitative digital EEG was done for all patients and interpreted by the researcher. This qualitative EEG was done to assess for possible apparent abnormalities in the EEG as epileptic activity and slowing.
7. *ELISA kits for the anti-NMDA antibody*: This was obtained from Bioassay Technology Laboratory, Cat. NO ED0534Hu. It is used for the qualitative detection of anti-NMDAR ab NR2A subunit in serum, plasma, cell culture supernates, and cell lysates. Serum samples collected from study participants were collected in acid citrate dextrose yellow tubes. They were allowed to clot for 20 min then centrifuged for 2000 RPM for 20 min. Samples were stored at − 30° to preserve them until use. Positive samples were those > 200 pg/ml, negative samples were those < 100 pg/ml and borderline samples were those between 100 and 200 pg/ml).
8. *Statistical analysis*: The collected data was revised, coded, tabulated and introduced to a PC using Statistical package for Social Science (SPSS 25). As regards *descriptive statistics:* mean, standard deviation (± SD), and range for parametric numerical data, while median and interquartile range (IQR) for non-parametric numerical data and frequency and percentage of non-numerical data were used. While for *Analytical statistics: Student’s t*-test was used to assess the statistical significance of the difference between two study group means. *Mann-Whitney’s test (U test)* was used to assess the statistical significance of the difference of a non-parametric variable between two study groups. *The chi-square test* was used to examine the relationship between two qualitative variables. *Fisher’s exact test* was used to examine the relationship between two qualitative variables when the expected count is less than 5 in more than 20% of cells. *p*-value: level of significance, was judged as follows: *p* > 0.05: nonsignificant (NS). *p* < 0.05: significant (S). *p* <0.01: highly significant (HS).

Children suspected to have ASD were referred to the primary investigator for assessment to decide whether to be included in the study. One hundred two patients were referred for possible enrollment in the study over the period from 1/10/2019 to 16/3/2020 (beginning of the lockdown due to the COVID-19 pandemic). Only eighty-seven were diagnosed as having ASD and completed all the study procedures and therefore were the only ones included.

The validity of the autism diagnosis was increased by 3 steps for enrollment. Children were first diagnosed clinically using the DSM-5 criteria by an expert psychiatrist. The second step was to administer the ADOS-2. Finally, children scoring lower scores on communication and social skills subscales on Vineland compared with other subscales were included while others scoring low scores on all Vineland subscales were excluded to minimize the inclusion of cases who were otherwise be diagnosed as having global developmental delay.

## Results

The study included eighty-seven children with ASD compared to sixty normal healthy controls. The mean age of the patient population in the study was 80.53 +\− 29.71 months old. This was matched to the mean age of the control group which was 79.45 +\− 29.95 months old (*t* = 2.944, *p* = 0.568).

The patient group included sixty-nine (79.31%) males and eighteen (29.69%) females matching the control group which included forty-eight (80%) males and twelve (20%) females (*X*^2^= 0.01, *p* = 0.919).

A test for the anti-NMDAR antibody came back negative for all the control group and was positive in twenty-three (26.4%) of the patient group which is a statistically significant difference (*p* = 0.0157). This is further described in Table 1.

**Table 1:** Sociodemographic data and presence of anti-NMDA receptor antibody in the control and patient groups of the study

Parameter		Group		Test of significance		
		Control (*N* = 60)	Case (*N* = 87)			
		Mean ± SD	Mean ± SD	Value	*p*-value	Sig.
		*N* (%)	*N* (%)			
Age- months		80.53 ± 29.71	79.45 ± 29.95	*t =* 2.944	0.568^(T)^	NS
Gender	Male	48 (80%)	69 (79.31%)	*X*^2^ *=* 0.01	0.919^(C)^	NS
	Female	12 (20%)	18 (20.69%)			
Anti-NMDA receptor antibody	Present	0 (0%)	23 (26.4%)		0.0157^(C)^	S
	Absent	60 (100%)	64 (73.6%)			

*SD* Standard deviation, *N* Number, *S* Significant, *NS* Not significant, ^(T)^Student’s *t*-test of significance (*t* = Student’s *t*-test value). ^(C)^Chi-Square test of significance (*X*^2^ = chi-square test value)

The patient group of the study had seven children (8%) with a mild level of autism-related symptoms as scored in ADOS-2, forty-three (49.4%) moderate level of autism-related symptoms, and thirty (42.5%) high level of autism-related symptoms as demonstrated in Table 2.

**Table 2:** Statistical correlation of anti-NMDAR antibody with developmental milestones in the patient group of the study

Parameter		NMDA		Test of significance		
		Negative	Positive			
		Mean ± SD	Mean ± SD	Value	*p*-value	Sig.
		*N* (%)	*N* (%)			
Gross motor milestones	Adequate	44 (68.75%)	23 (100%)	*X*^2^ *=* 9.33	0.002^(C)^	S
	Delayed	20 (31.25%)	0 (0%)			
Fine motor milestones	Adequate	33 (51.56%)	20 (86.96%)	*X*^2^ *=* 8.90	0.003^(C)^	S
	Delayed	31 (48.44%)	3 (13.04%)			
Speech stage	Preverbal	55 (85.94%)	14 (60.87%)		0.017^(F)^	S
	Phrase	9 (14.06%)	9 (39.13%)			
**Regression**	None reported	28 (43.75%)	7 (30.43%)	*X*^2^ *=* 1.248	0.264^(C)^	NS
	Present	36 (56.25%)	16 (69.57%)			
Age when regression was noticed—months		9 (0–24)	12 (0–24)	*U =* 677.0	0.554^(M)^	NS

*SD* Standard deviation, *N* Number, *S* Significant, *NS* Not significant, ^(T)^Student’s *t*-test of significance (*t* = Student’s *t*-test value). ^(C)^Chi-square test of significance (*X*^2^ = chi-square test value). ^(F)^Monte-Carlo Fisher’s exact test of significance. ^(M)^Mann-Whitney’s test of significance (*U* = Mann-Whitney’s test value)

Sixty-seven children (77%) of the patient group had adequate gross motor development. Fifty-three (60.9%) had adequate fine motor development. All children had delayed language development with sixty-nine (79.3%) having pre-speech or single words language level and eighteen (20.7%) had phrase speech. Fifty-two children (59.8%) reported regression of developmental milestones with mean age 21.5 months old +\− 9 months at which it was noticed with two peaks, between 8–12 months and 20–24 months old.

As regards findings from the stereotyped behavior scale, forty-four children (50.6%) in the patient group had a low degree of stereotyped behavior, thirty-seven (42.5%) with a below average degree, and six (6.9%) had an average degree of stereotyped behavior.

The probability to diagnose ASD (ASD%) as an endpoint for the Gilliam Autism Rating Scale (GARS) was that the average ASD% was 68.7 +\− 27.98%. The mean of the total calibrated score obtained from the Vinland scale was 35.09 +/− 12.32, with a median of 31 (IQR 31–44) and a range of 19–74. There is an overall tendency towards scores higher than 31 as evidenced by the mean being greater than the median and the IQR lowest range being the same as the median. The average age coefficient was 0.23 +/− 0.13 with a median of 0.18 and a range of 0.08–0.68. There is an overall tendency towards lower scores.

As regards the most affected subscale in Vineland, twenty-nine children had the communication subscale as the most affected subscale in Vinland social adaptive behavior scale, twenty-six had social interaction as the one most affected, and thirty-one had both communication and social subscales as the most affected.

Twenty-nine children had abnormal EEG findings representing 33.3% of the patient group of the study population. As regards the type of abnormality detected, eight children had epileptic discharge while twenty-one had abnormal slowing in the EEG.

As regards anti-N-methyl-d-aspartate (NMDA) receptor antibody (main study variable), twenty-three children tested positive for anti-NMDA receptor antibody (> 200 pg/ml) representing 26.4% of the study population.

The positive anti-NMDA receptor antibody was statistically significantly correlated with adequate gross (*X*^2^ *=* 9.33, *p* = 0.002) and fine (*X*^2^ *=* 8.90, *p* = 0.003) motor developmental milestones, better speech stage (in comparison to other autism populations) (*p* = 0.017) as shown in Table 2, more degree of severity in stereotyped behavior scale (*p* ≤ 0.001), higher total calibrated score in the Vinland Adaptive Behavior Scale (*p* ≤ 0.001), and abnormal EEG findings (*p* = 0.025) especially epileptic discharge (*p* ≤ 0.001) as shown in Tables 3 and 4. The positive anti-NMDA receptor antibody did not have any statistically significant correlation with other study variables.

**Table 3:** Statistical correlation of anti-NMDAR antibody with parameters obtained from clinical rating scales in the patient group of the study

Parameter		NMDA		Test of significance		
		Negative	Positive			
		Mean ± SD	Mean ± SD	Value	*p*-value	Sig.
		*N* (%)	*N* (%)			
**ADOS level of Autism related symptoms**	Mild	4 (6.25%)	3 (13.04%)	***X***^**2**^ ***=*** 3.786	0.151^(C)^	NS
	Moderate	29 (45.31%)	14 (60.87%)			
	High	31 (48.44%)	6 (26.09%)			
**Probability to diagnose ASD in GARS**		87 (65–89)	35 (19–84)	***U =*** 414.5	0.002^(M)^	S
**Stereotyped behavior** most affected	Sensory	30 (46.88%)	12 (52.17%)	***X***^***2***^***=*** 4.621	0.099^(C)^	NS
	Verbal	27 (42.19%)	5 (21.74%)			
	Motor	7 (10.94%)	6 (26.09%)			
Stereotyped behavior degree	Low	32 (50%)	12 (52.17%)		< 0.001^(F)^	S
	Below average	32 (50%)	5 (21.74%)			
	Average	0 (0%)	6 (26.09%)			
**Vineland’s** domain most affected	Communication	21 (32.81%)	8 (34.78%)		0.474^(F)^	NS
	Daily life	0 (0%)	0 (0%)			
	Social	19 (29.69%)	7 (30.43%)			
	Motor	0 (0%)	1 (4.35%)			
	Communication + Social	24 (37.5%)	7 (30.43%)			
Total calibrated score of Vinland Adaptive Behavior Scale		31 (26–31)	45 (39–51)	***U =*** 247.0	< 0.001^(M)^	S
Age coefficient		0.16 (0.13–0.27)	0.28 (0.2–0.4)	***U =*** 365.5	< 0.001^(M)^	S

*SD* Standard deviation, *N* Number, *S* Significant, *NS* Not significant, ^(T)^Student’s *t*-test of significance (*t* = Student’s *t*-test value). ^(C)^Chi-square test of significance (*X*^2^ = chi-square test value). ^(F)^Monte-Carlo Fisher’s exact test of significance. ^(M)^Mann-Whitney test of significance (*U* = Mann-Whitney’s test value)

**Table 4:** Statistical correlation of anti-NMDAR antibody with EEG findings in the patient group of the study

Parameter		NMDA		Test of significance		
		Negative	Positive			
		Mean ± SD	Mean ± SD	Value	*p*-value	Sig.
		*N* (%)	*N* (%)			
**EEG**	Normal	47 (73.44%)	11 (47.83%)	4.994	0.025	S
	Abnormal	17 (26.56%)	12 (52.17%)			
Finding of EEG	No	47 (73.44%)	11 (47.83%)	24.515	< 0.001	S
	Epileptic discharge	0 (0%)	8 (34.78%)			
	Slowing	17 (26.56%)	4 (17.39%)			

*SD* Standard deviation, *N* Number, *S* Significant, *NS* Not significant

## Discussion

To our knowledge, this is the first study to explore the relation between the presence of anti-NMDA receptor antibodies and ASD in a systematic way. Nearly a quarter of the patients’ group (26.4%) had tested positive for anti-NMDA receptor antibodies.

Previous research has shown that about 30–69% of ASD children have neuroinflammation or encephalitis [19]. Specifically, the so-called anti-brain autoantibody may damage fetal or children’s brain cells, eventually leading to children falling into an autistic or regressive state [7]. In the current study, only one-third of the patients proved to have anti-NMDA antibodies, this could be related to the type of sampling and the choice of the antibody to study.

Several autoantibodies have been reported to occur in the ASD population with varying frequency. There were many early studies investigating this possibility. About 58% were found to have anti-myelin basic protein [31]. Circulating antibodies towards neuronal and glial filament proteins have also been reported in autism [30]. Also, circulating antibodies against the caudate nucleus were found in sera of 49% of 68 autistic children [29].

Frye et al. [13], found that 75.3% of their studied ASD population have folate receptor autoantibodies. 62.5% of a studied ASD population had positive serum anti-neuronal antibodies and 74% tested positive for anti-ganglioside antibodies and both were correlated with more severe symptoms of autism [24, 25].

There were also reports of maternal anti-brain antibodies in mothers of autistic children [35]. It also has been reported that 25% of autistic children may have elevated anti-nuclear antibodies [26]. Increased serum anti endothelial cell antibody levels were observed in 63% of autistic children [4]. The rates reported in the current study (26.4%) are close to the lower range of other reported autoantibodies. This may be explained by the different courses that an anti-NMDA antibody pathology may take whether an acute and totally regressive course or a subacute and lesser regressive course than other antibodies.

A suggested further research in this area is to investigate the co-occurrence of these antibodies in the same population which may shed some light on the interaction between these autoantibodies and their combined effect.

The current study found that, adequate gross and fine motor development were correlated with positive anti-NMDA receptor antibodies. This may suggest that the anti-NMDA receptor antibody (if proven as a possible etiology to a subset of autism disorder) causes a milder type of brain affection in autism, or possibly due to partial recovery from anti-NMDAR encephalitis as a natural course of the illness [17]*.*

Previous research demonstrated that the degree of motor development predicted the subsequent development of language in children with autism [5]. Toe walking and age when first sitting predicted the severity of stereotyped behavior [33]. Both severity of stereotyped behaviors and better degree of language development had a statistically significant correlation with the positive anti-NMDA receptor antibody as shown in Table 3.

Better speech stage compared to the rest of the autism population (phrase speech rather than preverbal/single word) was correlated with having positive anti-NMDA receptor antibodies. Furthermore, the positive anti-NMDAR antibody was correlated with a more calibrated score and age coefficient on the Vineland adaptive behavior scale. The current finding contrasts with previous research that demonstrated persistent cognitive impairment of patients with NMDA encephalitis [12]. This could be related to the severity of the encephalitis, as none of the parents reported flu-like symptoms at the onset of disease, and different age and presentation of the current sample.

The basal ganglia have been linked to involuntary movements and stereotyped behaviors [11]. Also, several antigens as targets to autoantibodies have been linked to various basal ganglia pathology and its resulting disorders including DPPX: dipeptidyl-peptidase-like protein-6, GFAP: glial fibrillary acidic protein, mGluR1: metabotropic glutamate receptor 1, and NMDAR: N-methyl-d-aspartate receptors [16]. This may explain how the anti-NMDAR antibody is correlated with the severity of stereotyped behavior due to its action on the basal ganglia.

The kits used are directed towards the NR2A subunit of the NMDA receptor. NR2A and NR2B mRNAs are prominent in CA1 and CA3 pyramidal cells. NR2A is predominant in corticolimbic regions and is expressed mainly post-natal [3]. This may point out that the area in question may be the prefrontal cortex or part of the limbic system such as the anterior cingulate cortex.

From the integration of these data, we can conclude that the anti-NMDAR antibody affects the severity of, and is correlated to, stereotyped behaviors more than social interaction, and both more than the language domain in autism.

Positive anti-NMDAR was correlated with abnormal EEG findings, particularly with epileptic discharge rather than slowing. This agrees with the general notion that the NMDAR pathology is correlated with abnormal EEG findings [15], though EEG patterns of the delta brush were more commonly reported in this study than the epileptic discharge.

One of the important parameters that did not have a significant correlation with the presence of the anti-NMDA receptor antibody was the occurrence of regression in developmental milestones. This contrasts with what was initially hypothesized. This may point to the need for detection of anti-NMDAR antibodies earlier, e.g., in mothers or in amniotic fluid as previously suggested for other antibodies by [35]. The antibody may have been present from birth while exerting its effects at different stages or periods of brain development. Also, including a larger sample considering the acknowledged ratio between regression and non-regression groups of autism may enhance the statistical significance measurement of anti-NMDAR in autism. Moreover, recall bias cannot be excluded due to the cross-sectional stage sampling of the current study.

In comparing the clinical profile of the studies’ anti-NMDA receptor antibody with other autoantibodies, [13] did not find a correlation of folate receptor autoantibodies with developmental profile, occurrence of regression, type of regression, or EEG changes. Other studies correlated the presence of autoantibodies with more severity of ASD symptoms such as [13, 24]. This may seem to contradict the finding of this study where the positive antibody group had a better language level than the negative group. However, this may be explained by the notion that the effect of the anti-NMDA receptor antibody is related to its effects on the developing brain rather than a “quantitative” autoimmune pathology. Also, the more specific brain regions that this antibody acts upon as mentioned above that the receptor in question is concentrated more in certain brain regions.

Limitations of this study include a relatively small sample size which was due to premature termination of the study due to the COVID-19 pandemic. Also, the use of ELISA kits for the detection of NMDAR ab instead of the cell-based assay is due to higher expenses of the cell-based assay. The use of qualitative kits for anti-NMDA receptor antibodies was also a limitation, as a quantitative analysis would have provided better correlations with severity and other parameters. Other inflammatory markers in blood which could have been done, such as ESR (erythrocyte sedimentation rate), ANA (anti-nuclear antibody), and cytokine panel, again was not done due to expenses.

Future research in relation to this study may include functional brain imaging in correlation to the population with positive anti-NMDAR antibodies and prospective studies monitoring the severity of symptoms of the autism disorder with the level of anti-NMDAR antibody. Also, research targeting the presence of anti-NMDAR antibodies in sera of mothers with children with autism and research targeting the effect of immunological therapies such as steroids, IVIG, and plasmapharesis in children with autism testing positive for anti-NMDAR antibodies.

## Conclusions

There is a possibility of the presence of anti-NMDAR antibodies in the autism spectrum disorder population with certain characteristics, especially the stereotyped behaviors. This may enhance the trial of new treatment modalities targeting the immune system in at least 30% of the cases.

## Data Availability

The datasets used and/or analyzed during the current study are available from the corresponding author on reasonable request.

## Abbreviations

ADHD: Attention-deficit and hyperactivity disorder
ADOS-2: Autism diagnostic observation schedule-2nd edition
ANA: Anti-nuclear antibody
ASD: Autism spectrum disorder
BDNF: Brain-derived neurotrophic factor
COVID: Coronavirus disease-19
DNA: Deoxyribonucleic acid
DSM-5: Diagnostic and Statistical Manual, 5th edition
EEG: Electroencephalogram
ELISA: Enzyme-linked immunosorbent assay
ESR: Erythrocyte sedimentation rate
GARS: Gilliam Autism Rating Scale
HS: Highly significant
IQR: Interquartile range
MBP: Myelin basic protein
NMDA: N-Methyl-d-aspartate
NMDAR: N-Methyl-d-aspartate receptor
NS: Nonsignificant
PC: Personal computer
Pg: Picogram
RPM: Rotation per minute
S: Significant
SD: Standard deviation
SPSS: Statistical Package for Social Science
